# Reducing Home Labs for Pediatric Patients With Solid Tumors: Impact of a Novel Clinical Practice Guideline on Safety, Cost, and Quality of Life

**DOI:** 10.1002/cam4.71269

**Published:** 2025-09-26

**Authors:** Catherine B. Wall, Jill A. MacDonald, Riley Garland, Allison F. O'Neill

**Affiliations:** ^1^ Department of Nursing Dana‐Farber Cancer Institute/Boston Children's Cancer and Blood Disorders Center Boston Massachusetts USA; ^2^ Department of Quality and Patient Safety Dana‐Farber Cancer Institute/Boston Children's Cancer and Blood Disorders Center Boston Massachusetts USA; ^3^ Department of Pediatric Oncology Dana‐Farber Cancer Institute/Boston Children's Cancer and Blood Disorders Center and Harvard Medical School Boston Massachusetts USA

**Keywords:** guidelines, pediatric, quality, safety, solid tumors

## Abstract

**Introduction:**

For pediatric patients with solid tumors, the use of growth factors following chemotherapy evolved from daily filgrastim to pegylated filgrastim. Currently, post‐chemotherapy laboratory work is obtained to monitor for transfusion needs rather than count recovery. We implemented a clinical practice improvement project aimed at identifying the minimum number of labs required to assess transfusion needs, based upon disease and chemotherapeutic regimen, to design guidelines to safely reduce the number of home labs for targeted solid tumor diagnoses by 50% over 2 years.

**Methods:**

We retrospectively sourced data for a cohort of solid tumor diagnoses and chemotherapeutic regimens to determine the reasons why laboratory work was obtained. We subsequently developed new lab monitoring guidelines and conducted serial teaching sessions and prospective data monitoring post‐implementation.

**Results:**

Post‐implementation data analysis resulted in a reduction in home laboratory draws by 71%, exceeding our a priori set 50% goal. There were no adverse events. Estimated cost savings over 2 years, accounting for provider follow‐up, price per lab, and visiting nurse sessions, were $69,898. This reduction in home laboratory draws was interpreted, by decision tree analysis, to reduce the risk and costs associated with central line infections and improve quality of life.

**Conclusion:**

Our findings indicate that there is a cohort of solid tumor diagnoses that do not require twice weekly bloodwork to monitor for transfusion needs. Implementation of a novel clinical practice guideline resulted in a safe reduction in labs leading to optimized resource utilization, decreased costs, and perceived improvement in quality of life.

## Abstract 2

This clinical practice improvement project developed and safely implemented reduced lab monitoring guidelines for pediatric patients with solid tumors receiving common chemotherapy regimens.

## 1 Introduction

Patients treated for solid tumors with regimens known to result in neutropenia, independent of diagnosis and treatment regimen, historically received daily filgrastim as growth factor support. Standard of care at our institute dictated twice weekly lab monitoring to determine the timeline of filgrastim discontinuation after white blood cell recovery. Twice weekly labs became common practice for most patients with solid tumors at our institution independent of growth factor use. In multiple studies dating back to 2005, peg‐filgrastim was subsequently found to be safe and effective in pediatric patients [1, 2, 3]. As a result of these studies, our current standard has evolved to offer peg‐filgrastim to most patients receiving therapy for a solid tumor diagnosis. Peg‐filgrastim typically requires lab monitoring for transfusion needs alone as opposed to white blood cell count recovery. A consensus statement published in 2017 surrounding the use of peg‐filgrastim highlighted the less frequent lab draws to monitor absolute neutrophil count (ANC) as a distinct advantage to using this medication [4]. Despite an evolution in treatment approach and more consistent use of peg‐filgrastim in our patient population with solid tumors, we noticed institutionally that patients were still undergoing twice weekly labs. Twice weekly labs require more frequent line access or needle sticks, complex coordination of home care services or clinic scheduling, and devoted time for lab follow‐up. This level of follow‐up was proving onerous for patients, their families, and the provider team—most notably the nursing staff. A shortage in home care nurses further compounded scheduling challenges. Recently published data have revealed that shortages in home nursing most prominently affect pediatric patients compared with all other nursing fields [5, 6]. To address these challenges and the perceived excess in labs being obtained regularly, we embarked on an institutional clinical practice improvement project focused on identifying patients for whom twice weekly labs may not be clinically indicated. Our objective was to retrospectively evaluate the frequency of labs required to identify transfusion needs for patients with solid tumors across a range of standard therapies and to develop guidelines for the recommended frequency of required labs based upon the number of transfusions generally required per regimen or disease. Our aim was then to implement this guideline, follow patients prospectively and in real time, and determine whether we could safely reduce the number of home labs for targeted solid tumor diagnoses by 50%.

## 2 Methods

### 2.1 Context

This practice improvement initiative was conducted within a large academic pediatric oncology practice. At our institution, the pediatric oncology division is divided into four primary programs: solid tumor, hematologic malignancy, neuro‐oncology, and stem cell transplant. All patients with extracranial solid tumors are treated within the solid tumor program. Many of our patients qualify for home nursing services, that is, scheduling of home nurses, qualified in pediatric oncologic care, to perform lab draws in the patient's home. The samples are then brought to a local outside outpatient lab for processing. Our institution has dedicated nursing staff assigned to receive these results and facilitate coordination of care as needed. We elected to initiate this project to try to safely decrease the number of home labs obtained between chemotherapy cycles for pediatric patients receiving chemotherapy across a range of solid tumor diagnoses. There was support from programmatic and clinic leadership to implement this change. This project received exemption from the Dana‐Farber Cancer Institute Institutional Review Board.

We first performed a brief literature review to assess the scope of the problem and ascertain whether other sites were engaged in similar initiatives. We then launched a retrospective chart review to analyze data from two separate cohorts. Upon initial review across solid tumor diagnoses, we excluded common diagnoses/regimens if patients within the examined cohort required 15 or more transfusions (see Table 1 for diagnoses for which no changes were recommended). The initial cohort analyzed patients cared for with frequency at our Institute and included newly diagnosed cases of rhabdomyosarcoma, neuroblastoma, Wilm's tumor, and hepatoblastoma between July 2017 and August 2019. We repeated the same process with less frequently seen diagnoses including germ cell tumors, retinoblastoma, and soft tissue sarcomas. Patients who received daily filgrastim and patients who did not qualify for home nursing services (secondary to insurance disapproval, region, or family preference) were excluded. We reviewed the number of transfusions administered per diagnosis and treatment regimen. We also reviewed all laboratory values and clinical criteria utilized to determine the need for transfusion of either red blood cells or platelets.

**TABLE 1:** Solid tumor home lab monitoring guidelines by disease and regimen.

Disease	Regimens
**No Home Labs Between Cycles**	
Osteosarcoma	HD MTX (high‐dose methotrexate)
Rhabdomyosarcoma nonmetastatic	VAC/VI (vincristine, dactinomycin + cyclophosphamide/vincristine + irinotecan)
Wilm's tumor	DD4A (vincristine, dactinomycin + doxorubicin) or EE4A (vincristine + dactinomycin)
Retinoblastoma	VEC (vincristine, etoposide + carboplatin)
Germ cell	BEP/BEC (bleomycin, etoposide + cisplatin (P) or carboplatin (C))
**Once Weekly Labs**	
Hepatoblastoma	C5VD (cisplatin, 5‐FU, vincristine, + doxorubicin)
IR NB intermediate risk neuroblastoma	Up to 8 cycles containing combination of carboplatin, etoposide, cyclophosphamide, + doxorubicin
*Wilm's tumor (regimen M) slow pulmonary response	DD4A + cyclophosphamide + etoposide
*Soft tissue sarcoma	Ifosfamide + doxorubicin
**Twice Weekly Labs until Post Nadir**	
Ewing sarcoma	Compressed VDC/IE (vincristine, doxorubicin, + cyclophosphamide/ifosfamide + etoposide)
Hepatoblastoma	Dense dose cisplatin containing regimens
High risk (HR) neuroblastoma	HR (induction 5–6 cycles chemotherapy and autologous transplant)
Osteosarcoma	Cisplatin + doxorubicin/ifosfamide + etoposide
Rhabdomyosarcoma metastatic	VAC/VI (vincristine, dactinomycin + cyclophosphamide/vincristine + irinotecan)
Wilm's tumor anaplastic	VDC/ICE/VI (vincristine, doxorubicin +cyclophosphamide/carboplatin, ifosfamide + etoposide/VI)

*Note:* Depending on clinical status or clinical trial requirements, additional labs may need to be ordered.

^*^ No evaluable patients in the post‐guideline period (not included in Figure 2).

To accomplish this, a pediatric oncology registered nurse (RN), affiliated with the Solid Tumor program, first reviewed the electronic medical record of the identified patients and tabulated the number of transfusions a patient received. In an attempt to normalize transfusion needs across patients per disease and between cohorts, we calculated the mean number of lab draws performed.

### 2.2 Intervention

Based on this data, we developed lab monitoring guidelines categorized by disease and treatment regimen (Table 1). These guidelines, and the data supporting these guidelines, were reviewed and approved by the nursing director and clinical director of the solid tumor program. Educational sessions were performed at designated solid tumor faculty meetings, clinic conferences, and fellowship and nursing meetings. In November 2019, the guidelines were implemented for the initial cohort. The retrospective review of the subsequent cohort was performed in March 2020. Following the analysis of this data, these diagnoses were added to the guidelines in April 2020.

### 2.3 Study of the Intervention

To study the impact of our guideline implementation, we prospectively collected data from November 2019 through November 2021. We identified any new patients anticipated to receive the applicable standard of care therapy for the diagnoses listed in Table 1. During the course of therapy, charts were serially reviewed to assess compliance and safety with the lab guidelines. Particular focus was dedicated to the monitoring of transfusion needs to ensure no transfusions were missed due to a reduction in the number of labs obtained between cycles. When providers were noncompliant with the guidelines (i.e., excess labs were ordered), emails were sent to providers as a reminder of the intended lab frequency. Educational sessions continued throughout the intervention, and targeted emails were routinely sent to faculty, nursing, inpatient nurse practitioners (NPs), and outpatient NPs. In addition, the lab monitoring guidelines were laminated and posted throughout both the outpatient clinic and the inpatient unit. Two patients were excluded during the prospective data collection period due to the development of sinusoidal occlusive syndrome (SOS) and intensification of therapy, respectively.

### 2.4 Measures

The measure of the number of eligible patients successfully achieving a reduction in home labs was analyzed as follows. We calculated the mean number of home labs performed for each disease/treatment regimen in the prospectively followed cohort and compared this value with the retrospective cohort. We calculated a percent reduction in the mean number of labs drawn pre‐ and post‐implementation of guidelines normalized by the number of patients in each disease group. We subsequently performed a two‐tailed t‐test, assuming unequal variance, for reduction within each disease and an ANOVA to determine variance and assess reduction in labs across the entire cohort.

To estimate the cost savings during the prospective period, we assessed the absolute number of home labs performed in the retrospective cohort compared with the number of labs performed in the prospective cohort. We then calculated the net cost differential. The cost per home lab included the price to process a complete blood count with manual differential (CBCd) at Boston Children's Hospital ($131), the average visiting nurse costs of the three most utilized home nursing agencies ($135), and the average hourly pay rate in Massachusetts of an RN at the time of this project ($36.28 × 0.5 h = $18.14). We estimated that each lab required 30 min of time from a clinic nurse to review, formulate recommendations for transfusion, connect with the patient/family, and schedule necessary visits.

We used decision tree modeling to predict the cost saved for both lab frequency and for the potential of central line associated bloodstream infection (CLABSI), as well as determine the impact on quality of life as defined by inconvenience and pain/distress associated with line access. For the first analysis, we utilized the predicted rate of CLABSI in pediatric oncology patients to be 2.3 episodes per 1000 central line days [7, 8, 9] or approximately 0.85 episodes of CLABSI per year. The cost of a single CLABSI was estimated at $50,000 [10]. Rolling in the cost of labs performed (2X/week, 1X/week, or 1X/cycle) coupled with the potential for a CLABSI event, estimated to increase with an increase in line access, and the likelihood of needing labs 1X/week versus 1X/cycle, we were able to estimate predicted cost savings over the course of a year. The fractions assigned at each branch point indicate the anticipated proportion of patients affected. The second analysis assigned an anticipated quality adjusted life years (QALY) score to the inconvenience associated with lab draw frequency and pain/distress associated with recurrent central line access. There has been significant work surrounding the impact of procedures, in particular needle sticks, for pediatric patients with cancer and the anxiety generated from this intervention [11, 12]. This literature guided the proportion of children predicted to experience pain or distress related to needle sticks (i.e., 75%). There is less data to guide assignment of proportional impact on quality of life related to inconvenience or the need to be available for visiting nurse draws. These proportional values were therefore extrapolated from clinical experience and patient reports.

## 3 Results

**FIGURE 1:**
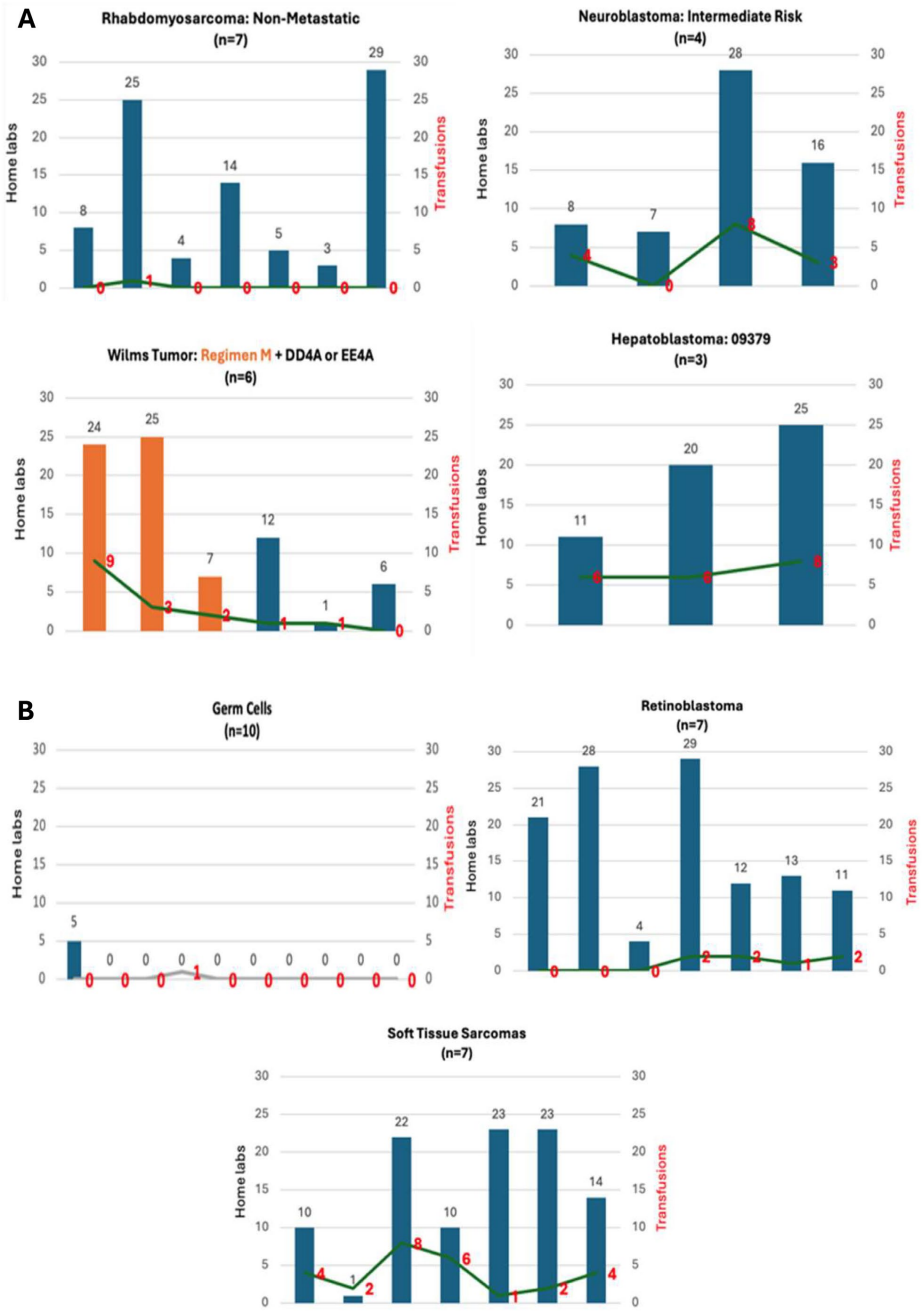
Number of home labs (y‐axis) and required transfusions (horizontal line) for individual patients by disease and chemotherapy regimen reflecting the results of our institutional retrospective data collection (initial cohort, A; subsequent cohort, B). Each bar represents an individual patient.

The retrospective chart review was performed on 44 patients diagnosed with a solid tumor and undergoing active treatment at the Dana‐Farber/Boston Children's Cancer and Blood Disorders Center from 2017–2019. Cohort demographics are detailed in Table 2. There were 20 patients included in the initial cohort (7 rhabdomyosarcoma, 4 neuroblastoma, 6 Wilm's Tumor, and 3 hepatoblastoma). In March 2020, 24 patients were reviewed in the subsequent cohort (10 germ cell tumor, 7 retinoblastoma, and 7 soft tissue sarcoma). Among these 44 patients, there were 504 home labs drawn (278 in the initial cohort, which included soft tissue sarcoma and Wilm's Regimen M, and 226 in the subsequent cohort). There were 87 total transfusions administered (52 in the initial cohort and 35 in the subsequent cohort). Transfusions were excluded if they were determined to be unrelated to the chemotherapy regimen (e.g., performed prior to or during a surgical procedure). Figures 1A (initial cohort) and 1B (subsequent cohort) depict the number of labs drawn per patient in contrast to the number of transfusions required, organized by disease and associated chemotherapeutic regimen. Data surrounding each of the 87 transfusions was analyzed to determine if the transfusion was triggered by the results of a home lab draw. It was determined that 18 out of the 87 transfusions were administered due to home lab draws. Given that there were no patients with soft tissue sarcoma or metastatic Wilms' in the prospective cohort, we eliminated values pertinent to these patients in the pre‐implementation cohort and utilized the following baseline data: 345 total labs drawn and 46 total transfusions administered.

**TABLE 2:** Baseline characteristics of patients.

	Retrospective cohort *N* = 44	Prospective cohort *N* = 43
Diagnosis		
Rhabdomyosarcoma	7	7
Neuroblastoma (IR)	4	4
Wilm's tumor	6	19
EE4A/DD4A	(3)	(19)
Regimen M	(3)	(0)
Hepatoblastoma	3	2
Soft tissue sarcoma	7	0
Germ cell tumor	10	7
Retinoblastoma	7	4
Gender		
Female	23	21
Male	21	22
Race		
White	30	24
African American	4	8
Asian	3	0
Other/declined	7	11
Age at Dx		
0–2 years	15	16
3–5 years	8	17
6–10 years	5	3
11–18 years	8	6
> 18 years	5	1
Total	44	43

After the new home lab monitoring guidelines were implemented, 43 patients were prospectively monitored from November 2019 to November 2021. During this timeframe, there were 99 home labs drawn. Of these 99 home lab draws, all but 7 were indicated per the guidelines. There were a total of 63 transfusions administered. Twelve of the transfusions were triggered by the results of a home lab draw. Of these 12, 6 were scheduled for clinical symptoms. The remaining six were patients that required once weekly home labs per our guidelines. In the prospective cohort, 5 patients had excess labs drawn (the 7 labs referenced above) that were not clinically indicated. Prompt reminders of the lab guidelines were sent to the patients' primary providers, effectively resulting in no further excess draws.

**FIGURE 2:**
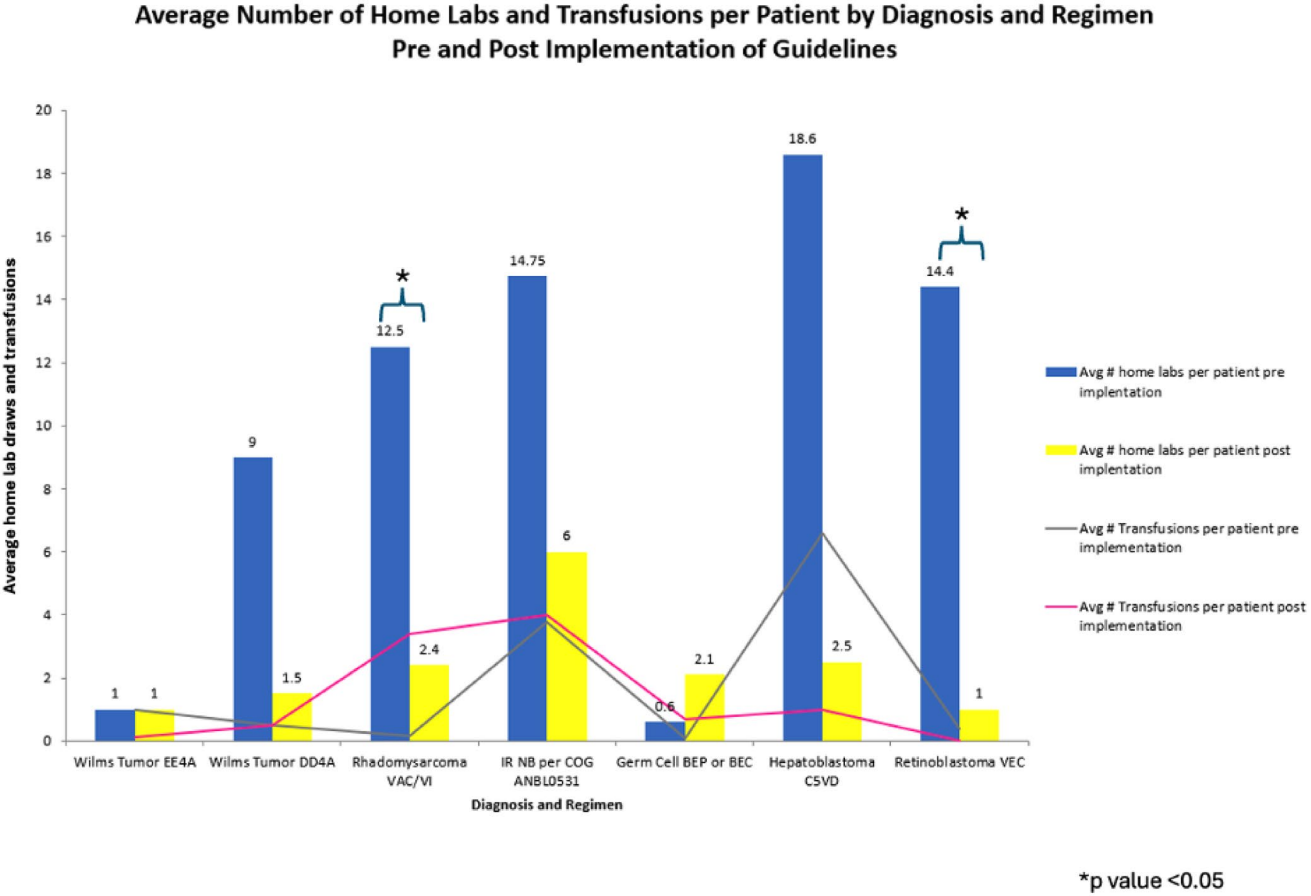
Average number of home labs per patient pre‐ (blue) and post‐ (yellow) implementation (y‐axis) and average number of transfusions per patient pre‐ (gray) and post‐ (red) guideline implementation (horizontal lines) by disease and chemotherapy regimen.

Implementation of the new lab guideline resulted in a 71% reduction in the number of home lab draws compared with historic data, exceeding the 50% goal of this project. The average number of home labs and transfusions per patient pre‐ and post‐guideline implementation is demonstrated in Figure 2. T‐tests demonstrated significance (*p* < 0.05) for only two diseases (rhabdomyosarcoma and retinoblastoma). ANOVA calculations demonstrated, as suspected, significant variance across diseases in the entire cohort (F statistic: 10.3) but less variation within each disease (F statistic: 1.3). While the ANOVA *p*‐value within each disease category was non‐significant (*p* = 0.38), likely reflecting the small number of patients within each disease cohort, the *p*‐value across the entire cohort demonstrated a significant reduction in overall labs (*p* = 0.02).

**FIGURE 3:**
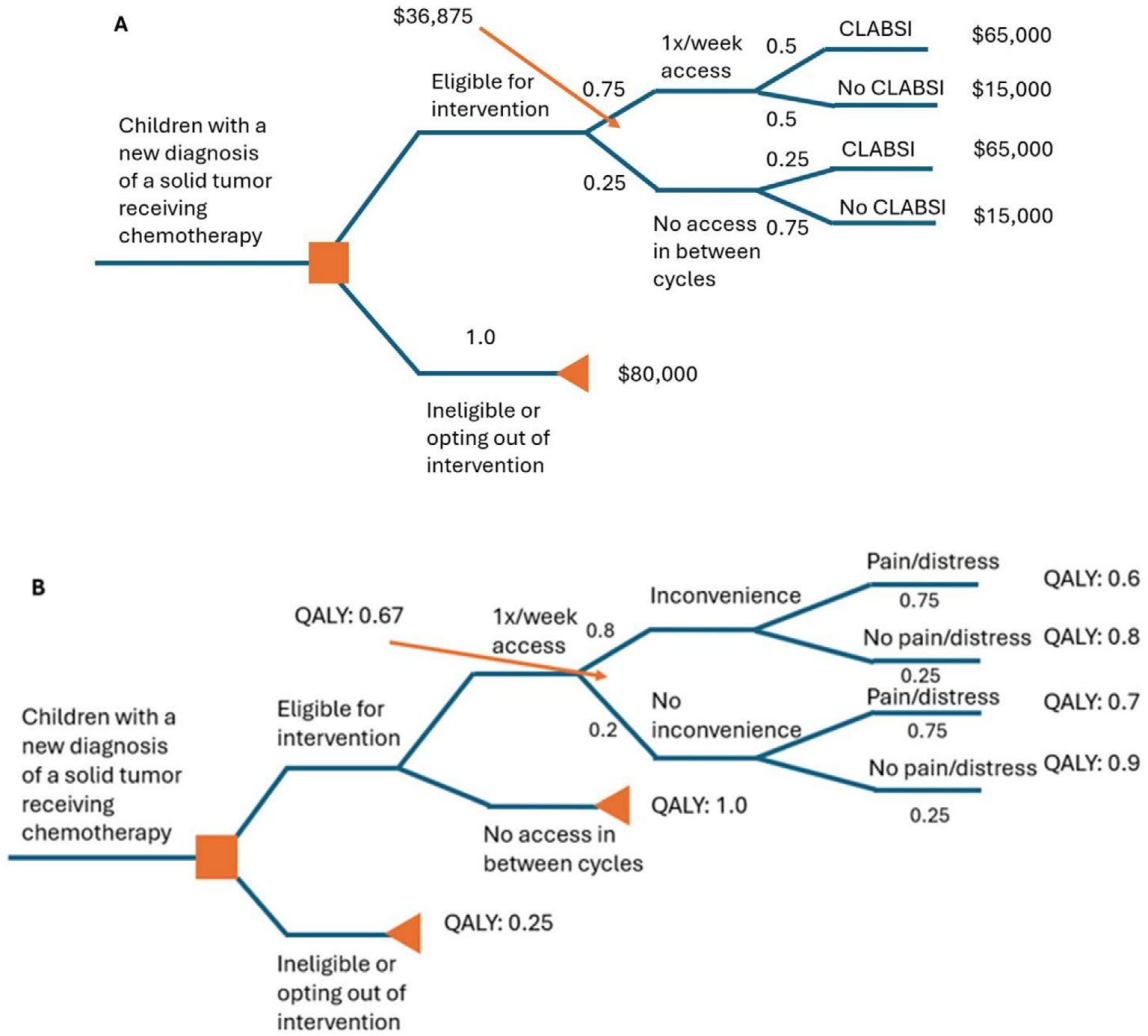
Decision tree modeling to predict the cost saved for both lab frequency and for the potential of central line associated bloodstream infection (CLABSI, A) as well as impact on quality of life as defined by inconvenience and pain/distress associated with line access (B). The fractions indicated at each branch point are representative of the anticipated proportion of patients affected. The orange arrows indicate back‐calculations, based on these proportions, of the anticipated impact on cost (A) or quality of life (B).

No patients presented with severe anemia or thrombocytopenia, concerning for clinical sequelae, due to a reduction in lab monitoring. There were two concrete benefits (cost and nursing time) resulting from this project as well as two modeled, predicted benefits (decreased risk for infection—and associated costs—and improved quality of life). Due to the reduction of 246 home lab draws post‐guideline implementation, the estimated cost savings over 2 years, accounting for oncology nursing and provider follow‐up, price per lab for processing, and visiting nurse sessions was $69,898. Estimating 30–60 min per home lab draw (time for travel, lab draw, and lab drop‐off), approximately 200–400 h of home nursing time was spared. Estimating 30 min of clinic nursing time per home lab draw (to interpret values and communicate results with the treating team and family), approximately 200 h of clinic nursing time was freed up for alternate focus. Utilizing decision tree analysis to estimate the impact of cost on CLABSI per year, we estimated over $40,000 in cost savings and over double the quality adjusted life years as measured by inconvenience to families in requiring they remain home for nursing visits as well as pain and distress (both anticipatory and associated with needle sticks) for each draw (Figure 3A,B).

## 4 Discussion

This clinical practice improvement project was initiated in response to the recognition that time spent by institutional nursing in necessary follow‐up to the substantive volume of home labs drawn in between chemotherapy cycles for pediatric patients with a solid tumor diagnosis was increasingly burdensome. Additionally, the project was conducted with the recognition that pediatric home nursing services have become increasingly limited. An initial retrospective chart review demonstrated that many patients undergoing frequent home labs between chemotherapy cycles never required transfusions. Subsequent design and implementation of a novel clinical practice guideline resulted in a safe reduction in home labs that surpassed a priori identified goals. By reducing the number of hours required for follow‐up on results, nursing and providers were available for other, more urgent needs. Of added value, we estimated significant cost savings and improvement in quality of life for patients undergoing this intervention. During our 2 years of prospective monitoring, there were no adverse effects associated with adherence to the new guidelines.

There were two elements that were crucial to the success of this implementation: provider buy‐in and dedicated personnel time. Presentation of this data across multiple disciplines was necessary to receive support for the newly created guidelines. The solid tumor program nursing champions who conceived of this project operationalized all steps committed to real‐time monitoring of all patients on active therapy to ensure that the guidelines did not lead to any adverse outcomes (i.e., patients presenting symptomatically due to a delay in labs and/or with critical counts requiring emergent transfusion). The proportion of home labs translating to a transfusion need post‐implementation (12 of 63 transfusions) suggests there may be further opportunity for lab reduction for a select cohort of patients.

At the time of completion of the prospective data collection, our pediatric oncology clinic transitioned to a disease‐specific oncology nurse navigator (ONN) model. This dedicated program‐specific group of nurses, with disease‐specific knowledge of the solid tumor patient population and protocols, will be a key factor in ensuring the lab monitoring guidelines will be continually implemented beyond this project's endpoint. The ONNs can also provide valuable insight into any possible updates to the guidelines as standards of care evolve and transfusion needs change. Additionally, the hospital's electronic medical record (EMR) will allow continual updates to chemotherapy order sets to include the recommended frequency of lab monitoring post‐chemotherapy cycles. This change will further ensure the permanency of this lab surveillance approach and guide individual providers when ordering specific chemotherapy regimens. Ultimately, as with any clinical practice initiative, clinical context remains the gold standard. Additional labs, outside of these guidelines, may be required to address clinical status or clinical trial requirements.

The primary limitation of this project surrounds the small sample size evaluated within each disease/regimen. During our prospective data collection, there were no patients diagnosed with soft tissue sarcomas treated with ifosfamide and doxorubicin or patients with Wilm's tumor treated with Regimen M (addition of cyclophosphamide/etoposide) per Children's Oncology Group (COG) AREN0553. Therefore, our guidelines could not be validated for these two populations. On the converse, our prospective cohort did allow us to validate recommendations for the majority of patients in our initial cohort. One of the 7 rhabdomyosarcoma patients in the prospective cohort developed veno‐occlusive disease leading to multiple transfusions. We included this patient to show that the primary team followed the guidelines; however, this contributed largely to the increase in transfusions post‐guideline implementation in this group. An additional limitation was that we could not include patients who did not utilize home nursing services for lab draws. These patients' lab draws were all performed in our clinic; when analyzing this data, it was difficult to ascertain whether labs were done for a clinical reason as opposed to standard twice‐weekly monitoring. Patients who did have labs drawn by home nurses had results scanned into a dedicated section in our EMR, making it easy to track and differentiate reason/timing for each timepoint. Despite these limitations, the data collected as a result of the project has supported full adoption of these guidelines and concrete clinical practice changes at the Dana–Farber/Boston Children's Cancer and Blood Disorders Center.

## 5 Conclusions

Based on this clinical practice initiative, the new laboratory guidelines have been accepted as standard of care at our Cancer Center for pediatric patients with a solid tumor diagnosis. This work has led to a decrease in vital resources and cost in an already burdened health care system and has contributed meaningfully to improving the quality of life for our patients. Incorporation into future chemotherapy order sets as well as educational training sessions for new nurses and providers will ensure ongoing sustainability.

The intentional development of these guidelines, based on COG regimens which are used widely across the country, will potentially allow implementation at other pediatric oncology centers. Future steps include continual updates to the guidelines as new standard chemotherapy regimens emerge and partnering with our hematologic malignancy and neuro‐oncology colleagues to pilot similar initiatives within their groups for standard chemotherapeutic regimens pertinent to their patient populations. On a broader scale, one can envision the applicability of this approach to other subspecialties for which patients are receiving therapy for chronic conditions. The scope of resource allocation and conservation is anticipated to be far‐reaching.

## Author Contributions

**Catherine B. Wall:** conceptualization (equal), formal analysis (equal), methodology (equal), writing – original draft (equal), writing – review and editing (equal). **Jill A. MacDonald:** conceptualization (equal), formal analysis (equal), methodology (equal), writing – original draft (equal), writing – review and editing (equal). **Riley Garland:** data curation (lead), formal analysis (supporting), visualization (equal). **Allison F. O'Neill:** conceptualization (supporting), formal analysis (equal), methodology (equal), supervision (lead), validation (lead), writing – review and editing (lead).

## Disclosure

Precis: This clinical practice improvement project developed and safely implemented reduced lab monitoring guidelines for pediatric patients with solid tumors receiving common chemotherapy regimens.

## Conflicts of Interest

The authors declare no conflicts of interest.

## Data Availability

The data that support the findings of this study are available from the corresponding author upon reasonable request.
